# Tuning of Redox Regulatory Mechanisms, Reactive Oxygen Species and Redox Homeostasis under Salinity Stress

**DOI:** 10.3389/fpls.2016.00548

**Published:** 2016-05-10

**Authors:** M. Sazzad Hossain, Karl-Josef Dietz

**Affiliations:** Department of Biochemistry and Physiology of Plants, Faculty of Biology, University of BielefeldBielefeld, Germany

**Keywords:** alternative oxidase, antioxidant enzymes, hydrogen peroxide, NADPH oxidase, salinity stress

## Abstract

Soil salinity is a crucial environmental constraint which limits biomass production at many sites on a global scale. Saline growth conditions cause osmotic and ionic imbalances, oxidative stress and perturb metabolism, e.g., the photosynthetic electron flow. The plant ability to tolerate salinity is determined by multiple biochemical and physiological mechanisms protecting cell functions, in particular by regulating proper water relations and maintaining ion homeostasis. Redox homeostasis is a fundamental cell property. Its regulation includes control of reactive oxygen species (ROS) generation, sensing deviation from and readjustment of the cellular redox state. All these redox related functions have been recognized as decisive factors in salinity acclimation and adaptation. This review focuses on the core response of plants to overcome the challenges of salinity stress through regulation of ROS generation and detoxification systems and to maintain redox homeostasis. Emphasis is given to the role of NADH oxidase (RBOH), alternative oxidase (AOX), the plastid terminal oxidase (PTOX) and the malate valve with the malate dehydrogenase isoforms under salt stress. Overwhelming evidence assigns an essential auxiliary function of ROS and redox homeostasis to salinity acclimation of plants.

## Introduction

Soil salinity is a major environmental stress that strongly impairs crop productivity and harvest quality in the world (Horie and Schroeder, 2004). Significant areas of the cultivated land in more than 100 countries are affected by salinity (Rengasamy, 2006). The quality of approximately 20% of the world's cultivated area and about 50% of the world's irrigated lands is affected by salinization (Sairam and Tyagi, 2004). Hence, soil salinity poses a serious threat to crop yield and future food production. Plant responses and tolerance mechanisms to salt stress are a major topic of plant research (Munns and Tester, 2008). In general, high salt concentrations induce ionic imbalances, osmotic stress and oxidative damage (Zhu, 2001). Glycophytic plants under salt stress conditions exhibit slow growth, wilting and eventually death (Parida et al., 2004). To survive under stress condition, plants respond and adapt with complex mechanisms that include developmental, morphological, physiological and biochemical strategies (Taji et al., 2004; Acosta-Motos et al., 2015) addressing ion homeostasis, osmolyte biosynthesis, compartmentation of toxic ions, and reactive oxygen species (ROS) scavenging systems (Stepien and Klobus, 2005; Flowers and Colmer, 2008). Many genes involved in membrane transport, signal transduction, redox reactions and other processes have been identified and characterized (Inan et al., 2004; Zhang et al., 2008). However, the quantitative contribution of the various molecular mechanisms, their qualitative interactions and the integrated functional network underlying plant tolerance to salt stress remain to be determined.

Cell metabolism generates reactive oxygen species (ROS) at low rates as normal side product. Salinity stress often enhances the generation of reactive oxygen species (ROS). This may lead to metabolic disorders, cellular damage, and premature senescence or necrosis (Møller et al., 2007; Jaleel et al., 2009; Miller et al., 2010; Habib et al., 2016). Excessively accumulating ROS may react with suitable targets such as nucleic acids, proteins, lipids and chlorophyll. The main ROS include non-radical molecules like singlet oxygen (^1^O_2_) and hydrogen peroxide (H_2_O_2_), as well as free radicals such as superoxide (${\text{O}}_{2}^{•-}$) and hydroxyl radicals (^•^OH) (Azevedo Neto et al., 2008). Besides their harmful effects ROS act as signaling molecules that regulate plant development, biotic and abiotic stress responses (Mittler et al., 2004). Recent research and considerations have focused on ROS metabolism (Noctor et al., 2014), sensory and signaling networks (Dietz, 2008; Miller et al., 2010; Suzuki et al., 2012; Baxter et al., 2014), as well as the cross-talk with other signaling pathways (Suzuki et al., 2012; Noctor et al., 2014).

**Figure 1:**
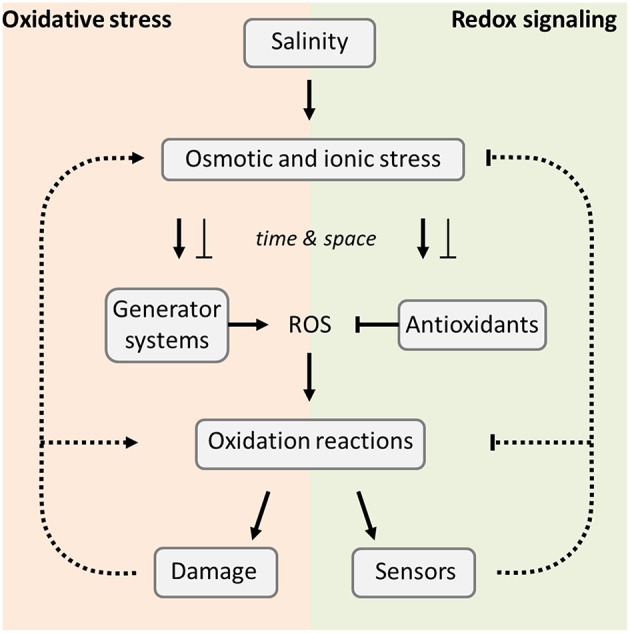
**Overview of salinity-induced stress effects**. The primary stress effects are osmotic and ionic imbalances which affect the ROS generator and antioxidant systems. This effect may be positive or negative as indicated and discussed. Their relative activities determine the ROS levels, as well as the subsequent redox reactions which are used in signaling **(right hand side)** or, if excessive, cause oxidative damage which enhances the stress effects **(left hand side)**.

The seemingly negative consequences of excess ROS accumulation like lipid peroxidation, oxidation of proteins, damage of nucleic acids, enzyme inhibition, and activation of programmed cell death (PCD) are also linked to signaling since the reaction products transmit information to downstream events (Figure 1; Mishra et al., 2011; Srivastava and Dubey, 2011). The steady state ROS levels depend on the rates of generation and decomposition (Figure 1). Three levels of specificity need to be considered at the level of ROS, their chemical reactivity, as well as their temporal and spatial accumulation pattern. A network of low molecular mass antioxidants and antioxidant enzymes, redox input elements, redox transmitters, redox target proteins and redox sensors orchestrate the readjustment of redox homeostasis and redox-dependent response (Azevedo Neto et al., 2008; Dietz, 2008; Gill and Tuteja, 2010). To understand the redox and ROS balance under salinity we have to consider (i) the ROS generator systems, (ii) the antioxidant defense system, and (iii) the redox regulatory network.

The best studied component is the antioxidant system which includes gene families encoding superoxide dismutases (SOD), catalases (CAT), guaiacol peroxidases (POX), the ascorbate-glutathione (ASC-GSH) cycle enzymes [ascorbate peroxidases (APX), monodehydroascorbate reductases (MDHAR), dehydroascorbate reductases (DHAR), glutathione reductases (GR)], glutathione peroxidases (GPX), peroxiredoxins (PRX) and glutathione S-transferases (GST) (Mittler et al., 2004; Azevedo Neto et al., 2008; Munns and Tester, 2008). These antioxidant enzymes are targeted to specific subcellular compartments and reveal stress-specific modulation of their expression as compiled e.g., by Mittler et al. (2004) for *A. thaliana*.

Intensive research on salinity has addressed the ROS generator systems which in a regulated or conditional, but partly less targeted manner control the release of ROS. Metabolic pathways like photorespiration and membrane-associated enzymes determine the rate of ROS generation in plant cells; the NADPH oxidase (respiratory burst oxidase homolog: RBOH) (Keller et al., 1998), mitochondrial alternative oxidase (AOX) (Considine et al., 2002) and plastid terminal oxidase (PTOX) (Stepien and Johnson, 2009). Additional layers of defense and regulation modulate the redox state of the cell and control the subcellular redox balance. Malic acid and oxaloacetic acid represent a redox pair of metabolites linked by malate dehydrogenases (MDH), e.g., the NADP-MDH in the chloroplast. Exchange of malic acid and oxaloacetic acid by membrane transport among compartments allows for indirect transfer of reducing equivalents. This process is known as malate valve (Scheibe et al., 2005). The tightly regulated plastid NADP-MDH controls the export of excess reducing power from the photosynthesizing chloroplast and thereby relieves overreduction of the photosynthetic electron transport chain and thus generation of ROS.

The significance of these enzymes and mechanisms in salt tolerance is still a matter of controversy. Often, regulatory patterns are difficult to interpret; e.g., upregulation of antioxidant enzymes may represent the response to manifested oxidative stress. On the other hand, upregulation of antioxidant enzymes may be considered as proactive acclimation response which results in lower ROS levels and higher tolerance to oxidative stress. Consequentially high antioxidant enzyme activities have been associated with salt tolerance as well as salt sensitivity. With that said, this review aims to provide a critical update on redox regulation, oxidative stress and their implications in salt stress acclimation and damage development.

## Generation of ROS under salinity stress in plant

Salt stress interferes with carbon metabolism and thereby fosters ROS generation. Stomatal conductance decreases during salt stress and lowers transpiration. The stomatal movement is linked to ion redistribution, alkalization and ABA accumulation (Geilfus et al., 2015). Restricted gas exchange limits CO_2_ uptake, lowers intercellular CO_2_ concentration and CO_2_ availability for the Calvin cycle. As a consequences the pool of oxidized NADP^+^ (as final electron acceptor at PSI) is depleted and electrons are transferred to O_2_ to generate ${\text{O}}_{2}^{•-}$ (Mehler, 1951). Following non-enzymatic or enzymatic dismutation by superoxide dismutase (SOD), H_2_O_2_ can be converted to the extremely reactive hydroxyl radicals (^•^OH) in the Fenton-/Haber-Weiss-reaction. Insufficient energy dissipation in photosynthesis causes formation of ^1^O_2_ from triplet chlorophyll (Chl) especially in the reaction center of photosystem II (Krieger-Liszkay, 2005). The decrease in the CO_2_/O_2_-ratio in the mesophyll enhances photorespiration in C3-plants and stimulates H_2_O_2_ generation in the peroxisome (Wingler et al., 2000; Ghannoum, 2009). Photorespiration accounts for over 70% of the H_2_O_2_ production under osmotic stress (Noctor et al., 2002). Early studies on respiratory electron transport (RET) reported increased rates of electron transfer to O_2_ and thus of respiratory O_2_ consumption under salt stress (Fry et al., 1986; Moser et al., 1991). ${\text{O}}_{2}^{•-}$ is generated when RET is overreduced. The generated ${\text{O}}_{2}^{•-}$ is dismutated to H_2_O_2_ which is subsequently reduced to water by catalases, class I peroxidases (APXs), class III peroxidases (POXs) and thiol peroxidases. Salinity stress activates the cell membrane-bound RBOH (Rejeb et al., 2015a; Tsai et al., 2005) and the apoplastic diamine oxidase (Waie and Rajam, 2003). Both mechanisms contribute to the generation of ROS in the apoplastic space. In other cases RBOH activity was inhibited under salt stress (Rodríguez et al., 2009). In salt-stressed maize leaves (150 mM NaCl in hydroponics), levels of apoplastic spermidine and spermine increased several times and the apoplastic polyamine oxidase allowed for converting the polyamines to 1,3-diaminopropane and H_2_O_2_ (Rodríguez et al., 2009). This mechanism enables cell wall loosening by generation of ^•^OH and sustains leaf blade growth even if RBOH is inhibited (Rodríguez et al., 2009). Other ROS-generating enzymes include oxalate oxidase and amine oxidase. In context of salinity much work has focused on RBOH-like enzymes, the major enzymatic route of ROS synthesis in plant cells (Sharma et al., 2012), which will be discussed below in more detail.

**Figure 2:**
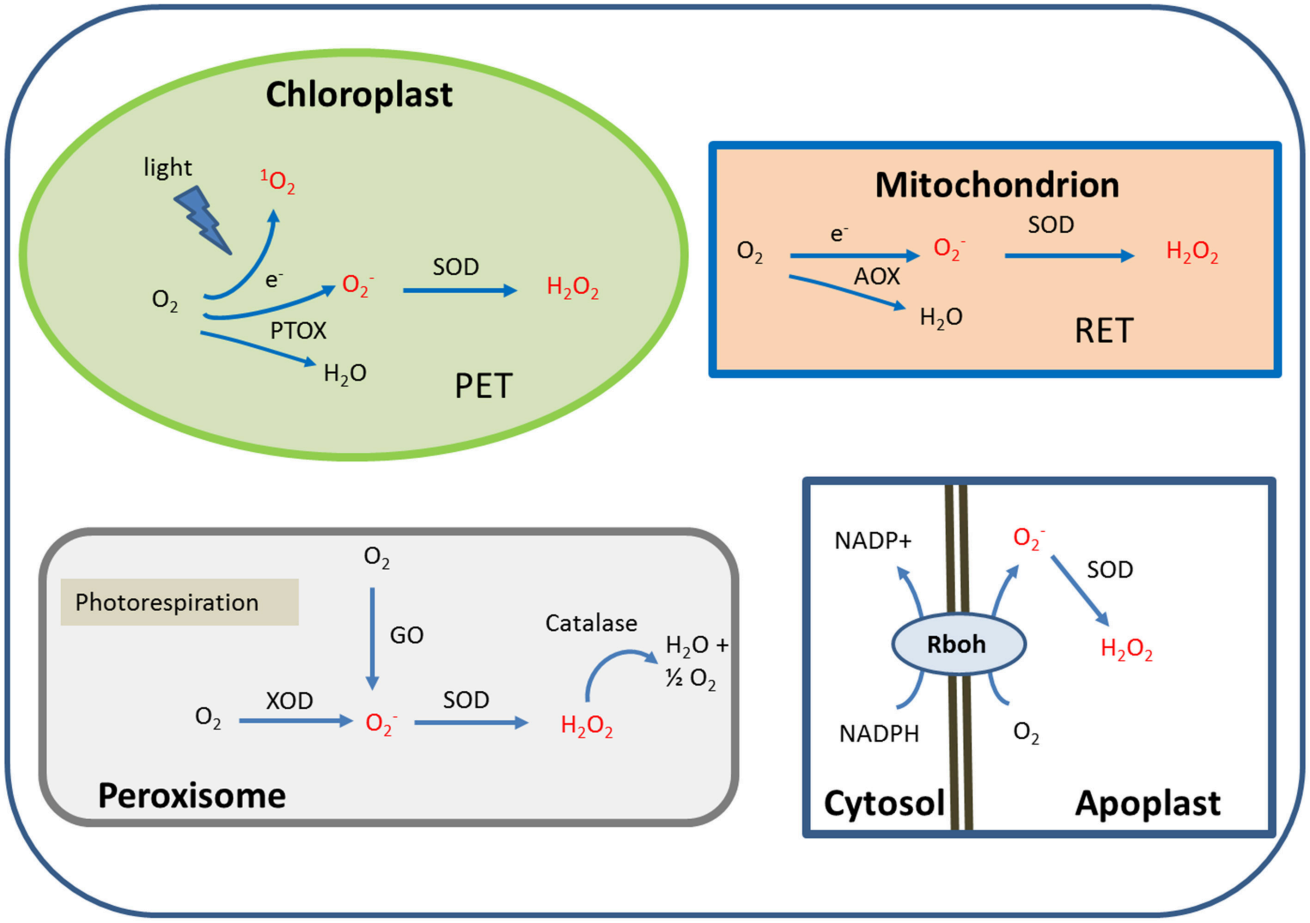
**Simplified scheme illustrating the predominant ROS generation sites in plant cell under salinity stress**. These sites are photosynthesis in chloroplasts, respiration in mitochondria, photorespiration in peroxisomes and NADH oxidation at the plasma membrane. AOX, alternative oxidase; GO, glycolate oxidase; PET, photosynthetic electron transport; PTOX, plastid terminal oxidase; RBOH, respiratory burst oxidase homolog; RET, respiratory electron transport; SOD, superoxide dismutase; XOD, xanthine oxidase.

Under salinity stress each type of organelle employs different mechanisms of ROS production. The subcellular and cellular sites of ROS production decisively determine their signaling action. Thus, ROS are produced in particular in the PET of chloroplasts, the RET in mitochondria, various oxidases in peroxisome and the NADPH oxidase (RBOH) in the plasma membrane (Figure 2). In addition reactions in the endomembrane system and the apoplast/cell wall also contribute to ROS generation (Table 1). Localized production in specific micro-compartments and the buffering action of multiple antioxidant mechanisms fine-tune the concentrations of ROS at particular sites of the cell under salt stress. Such mechanisms could delimit the response to localized “hot-spots.”

**Table 1:** **Generation sites and origin of reactive oxygen species (ROS) in plants**.

**Location**	**Key sources of ROS in cell**	**References**
Chloroplast	PET (PSI, PQ, and PSII) Chlorophyll pigments	Elstner, 1991; Cleland and Grace, 1999
Mitochondrion	Complexes of RET Enzymes, e.g., aconitase, 1-galactono-γ lactone dehydrogenase (GAL)	Andreyev et al., 2005; Rasmusson et al., 2008
Peroxisome	Glycolate oxidase (GO), fatty acid β-oxidation, flavine oxidases, xanthine oxidase (XOD), peroxisomal NADPH: cytochrome P450 reductase and ETC composed of a flavoprotein, NADH and Cyt b	López-Huertas et al., 1999; Baker and Graham, 2002
Plasma membrane	NADPH oxidase and menadione (quinone reductase)	Heyno et al., 2011
Apoplast	Cell wall-associated oxalate oxidase (germine) and amine oxidase-like enzymes	Wojtaszek, 1997; Cona et al., 2006
Cell wall	Cell-wall-associated peroxidase in the presence of NADH and diamine oxidases	Gross, 1977; Martinez et al., 1998
Endoplasmatic reticulum	NAD(P)H-dependent electron transport involving Cyt P450	Mittler, 2002

## Scavenging of ROS under salinity stress in plant

Superoxide dismutase (SOD) belongs to the group of metalloenzymes and functions as an important enzyme in the first line of antioxidant defense. MnSOD, Cu/ZnSOD and FeSOD dismutate ${\text{O}}_{2}^{•-}$ into H_2_O_2_ and O_2_ (Rios-Gonzalez et al., 2002; Tuna et al., 2008). Elevated SOD activity often appears to enhance plant tolerance to oxidative stress (Gupta et al., 1993). ^1^O_2_ can be quenched by β-carotene or α-tocopherol, but also can react with the D1 protein of photosystem II as a sensitive protein target (Krieger-Liszkay, 2005). Among the antioxidant enzymes, catalase (CAT) was discovered first and dismutates two molecules of H_2_O_2_ into water and oxygen. Peroxidases are classified as heme or thiol (or selenol) peroxidases and reduce H_2_O_2_ at the expense of an alternative electron donor (Dietz, 2016). Thiol peroxidases use thiol electron donors such as thioredoxin (TRX), glutaredoxin (GRX), glutathione or in rare cases ascorbate to convert H_2_O_2_ to H_2_O_._ Ascorbate peroxidase (APX) catalyzes the primary step in the classical water-water cycle (Asada, 1992) where DHAR and GR, a flavoenzyme which contains an essential disulfide group, catalyze the regeneration of ascorbate from dehydroascorbate using glutathione and finally NADPH as reductants. The regeneration of ascorbate from monodehydroascorbate is also carried out by MDHAR using NADH as reducing power. GPX, some GST and PRX reduce H_2_O_2_ and organic hydroperoxides through ascorbate-independent thiol-mediated pathways using nucleophiles such as GSH, thioredoxin (TRX) or glutaredoxins (GRX) (Asada, 1992; Dietz et al., 2006; Noctor et al., 2014). Transcript levels of TRX, Mn-SOD, AOX, and PRXIIF increased under short-term salinity conditions (Marti et al., 2011). The mitochondrial PsTRXo1 increases in pea leaves in response to long-term salinity (Marti et al., 2011). PsTRXo1 and PRXIIF provide the cell with a mechanism to protect mitochondria from oxidative stress together with Mn-SOD and AOX. Mitochondrial TRX-o1, PRXIIF and sulfiredoxins participate in the establishment of salt tolerance through a fine-regulated mechanism involving the post-translational modifications of *S*-glutathionylation and *S*-nitrosylation (Lázaro et al., 2013).

The link between salt tolerance and increased activities of antioxidant enzymes has frequently been established (Zeng et al., 2003; Liu et al., 2011; Table 2). Stepien and Klobus (2005) compared oxidative stress in salt-stressed wheat and maize by assessing lipid peroxidation and activities of antioxidant enzymes. Maize suffered less oxidative stress than wheat. The constitutive and salt stress-inducible activities of SOD, APX and GR were higher in maize than in wheat. The authors suggested that the higher tolerance of maize is based on two mechanisms, namely lower ROS production in C4-photosynthesis and higher activities of the antioxidant enzymes. Photorespiration is strongly induced by salt stress in C3 but not in C4 and CAM plant (Cushman and Bohnert, 1997). *Flaveria, Alternanthera, Parthenium, Panicum* and *Moricandia* species are some species with C3-C4 intermediate photosynthesis which are able to efficiently recycle photorespired CO_2_ which lowers the rate of photorespiration (Monson et al., 1984; Devi and Raghavendra, 1993). C3 species examined had high intrinsic levels of photorespiration whereas the C3-C4 intermediate species (primarily by refixing photorespired CO_2_), C4-like and C4 species (via selective localization of ribulose-1,5-bisphosphate carboxylase in bundle sheath cells and operation of a CO_2_ pump via the C4 pathway) maintained low apparent rates of photorespiration (Dai et al., 1996). In another study, Rios-Gonzalez et al. (2002) reported higher activities of GR, SOD, POD and CAT in sunflower leaves than in maize under salt stress. C4-like *Flaveria brownie* and C4 *Flaveria bidentis* are able to prevent oxidative damage by stress by increased enzymatic and non-enzymatic antioxidants, as compared to C3 (*Flaveria robusta*) and C3–C4 intermediate (*Flaveria anomala*) (Uzilday et al., 2014). Higher water use efficiency of C4 plants should support growth on saline soil. However, it appears too early to generalize on a possible advantage that C4 plants possibly have since many other traits participate in salinity tolerance a priori.

**Table 2:** **Antioxidant enzymes reported to be regulated in plants under salinity stress**.

Antioxidant enzymes^*^	**Plant species**	**References**
**SOD**, **CAT**, **GPX**, **APX**, **GR**, **MDHAR, DHAR**	*Oryza sativa*	Mishra et al., 2013
**CAT**, **SOD, GR**	*Olea europaea*	Valderrama et al., 2006
**GPX**	*Oryza sativa*	Mittal and Dubey, 1991
**APX, MDHAR, DHAR, GR**	*Oryza sativa*	Hossain et al., 2013
**SOD**	*Triticum aestivum*	Borzouei et al., 2012
	*Oryza sativa, Avicennia marina*	Prashanth et al., 2008
	*Nicotiana tabacum*	Van Camp et al., 1996
	*Oryza sativa*	Tanaka et al., 1999
	*Cakile maritime*	Ellouzi et al., 2011
**GR**, **SOD**, **POX, CAT**	*Helianthus annuus, Zea mays*	Rios-Gonzalez et al., 2002
**SOD**, **APX, DHAR**	*Nicotiana tabacum*	Lee et al., 2007
**APX, GR**	*Oryza sativa*	Tsai et al., 2005
SOD, APX, GR	*Triticum aestivum*	Stepien and Klobus, 2005
**SOD**, **APX, GR**	*Zea mays*	Stepien and Klobus, 2005
**CAT**, **APX, GR**	*Arabidopsis thaliana*	Rejeb et al., 2015a
**APX**	*Nicotiana tabacum*	Badawi et al., 2004; Li et al., 2009; Sun et al., 2010
	*Pisum sativum, Lycopersicon esculentum*	Wang et al., 2005
	*Hordeum vulgare*	Shi et al., 2001
	*Arabidopsis thaliana*	Lu et al., 2007
	*Ipomoea batatas*	Lin and Pu, 2010
**DHAR, GR**, MDHAR, APX, SOD	*Pisum sativum*	Hernández et al., 2001
**CAT**, **POX**, **APX, GR**, SOD	*Echinochloa crusgalli*	Abogadallah et al., 2009
**MDHAR**	*Nicotiana tabacum*	Eltayeb et al., 2007
**DHAR**	*Lotus japonicas*	Rubio et al., 2009
**GR**	*Nicotiana tabacum)*	Aono et al., 1993
	*Populus species*	Foyer et al., 1995
**SOD**, **CAT**, **APX, MDHAR**	*Lycopersicon esculentum*	Shalata et al., 2001
**SOD**, **CAT, APX**	*Gossypium hirsutum*	Luo et al., 2013
**POX**, CAT, SOD	*Vigna unguiculata*	Cavalcanti et al., 2004
**NOX/RBOH**	*Arabidopsis thaliana*	Sakamoto et al., 2008; Ma et al., 2012
NOX/RBOH	*Glycine max*	Song et al., 2012
NOX/RBOH	*Brassica juncea*	Srivastava et al., 2015
NOX/RBOH	*Sesuvium portulacastrum*	Srivastava et al., 2015
**RBOHD/F**	*Arabidopsis thaliana*	Ma et al., 2012
**RBOHF**	*Arabidopsis thaliana*	Jiang et al., 2012
**AOX**	*Citrus sinensis (cvs. Carvalhal tangor)*	Ferreira et al., 2008
	*Glycine max*	Hilal et al., 1998
	*Arabidopsis thaliana*	Smith et al., 2009; Wang et al., 2010
	*Hordeum vulgare*	Jolivet et al., 1990
**OsAOX1a**	*Oryza sativa*	Li et al., 2013
**OsAOX1b**	*Oryza sativa*	Li et al., 2013
OsAOX1c	*Oryza sativa*	Li et al., 2013
**NADH-MDH**	*Oryza sativa*	Kumar et al., 2000
	*Mesembryanthemum crystallinum*	Cushman, 1993; Gawronska et al., 2013
	*Arabidopsis thaliana*	Hebbelmann et al., 2012
**PTOX**	*Thellungiella halophila*	Stepien and Johnson, 2009
PTOX	*Arabidopsis thaliana*	Josse et al., 2003; Stepien and Johnson, 2009
	*Oryza sativa*	Kong et al., 2003
	*Haematococcus pluvialis*	Wang et al., 2009
	*Thellungiella salsuginea*	Wiciarza et al., 2015

* *Bold: upregulated under salt stress, underlined: down-regulated, normal: unchanged*.

A higher redox status of antioxidants and the coordinated increase in SOD, CAT, GPX, APX, and GR activities was suggested by Mishra et al. (2013) to serve as the major determinants of salt tolerance in Indica rice seedlings. In this study, the activity of CuZn-SOD, APX, GPX, CAT, MDHAR, DHAR, and GR increased in the salt tolerant cultivar like in salt-sensitive seedlings at moderate salinity of 7 dS m^−1^ NaCl. In contrast, the activity of GPX, CAT, MDHAR, DHAR, and GR decreased with higher salinity of 14 dS m^−1^ NaCl in the sensitive genotype. Cultivars with distinct salt sensitivity allow exploring involved mechanisms. Pusa Basmati-1 is highly salt sensitive, while Pokkali displays moderate salt tolerance. In parallel the activity of the ROS scavenging CAT and the levels of antioxidants like ASA and GSH are increased and concomitantly the membrane damage as judged from lipid peroxidation and H_2_O_2_ levels are lower in Pokkali compared to Pusa Basmati-1 (Vaidyanathan et al., 2003). Transcript regulation of peroxisomal APX (HvAPX1) correlates with salt stress (Shi et al., 2001). Lin and Pu (2010) studied the involvement of ROS scavenging enzymes in tolerant and sensitive sweet potato under salinity. The activity increase of cAPX, mAPX and chlAPX 24 and 48 h after exposure to 450 mM NaCl was higher in the salt stress-tolerant genotype than in the sensitive ones. Overall induction and maintenance of a strong antioxidant defense frequently correlates with enhanced salt tolerance.

**Figure 3:**
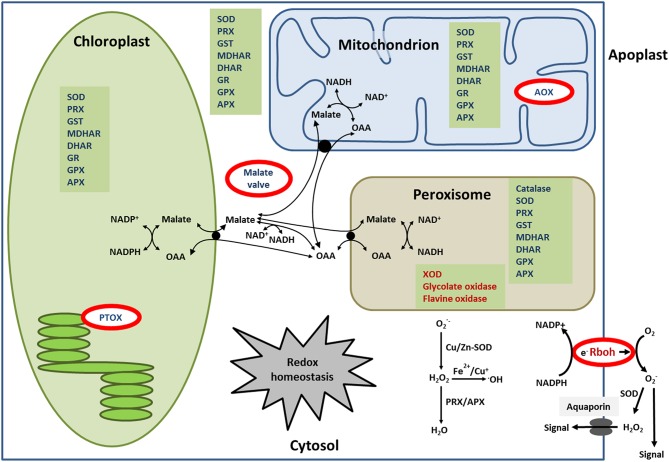
**Overview of the antioxidant systems and the malate valve**. This figure shows the distribution of the various antioxidant defense systems and the function of the malate valve as mechanism to equilibrate reductive power between organelles.

Likewise genetic fortification of antioxidant levels has been shown to enhance salinity tolerance by decreasing the oxidative stress. Transgenic tobacco overexpressing cytosolic AtMDAR1 exhibited up to 2.1-fold higher MDAR activity and 2.2-fold higher levels of reduced AsA than non-transformed control plants and concomitantly the tolerance to salt stress was enhanced (Eltayeb et al., 2007). Likewise, transgenic Arabidopsis over-expressing rice cytosolic OsAPXa/b exhibited increased salt tolerance compared to wild-type (Lu et al., 2007). A similar improvement in salt stress tolerance was observed in transgenic tobacco expressing the AtcAPX gene (Badawi et al., 2004) or the *Solanum lycopersicum* tAPX (SltAPX) (Sun et al., 2010). Transgenic tobacco simultaneously expressing CuZnSOD, APX, and DHAR in the chloroplast tolerated 100 mM NaCI without developing salt-induced injury observed in wild type (Lee et al., 2007). Prashanth et al. (2008) applied salt stress to indica rice var Pusa Basmati-1 overexpressing cytosolic Cu/ZnSOD from the mangrove *Avicennia marina*. The heterologous expression conferred salinity stress tolerance in hydroponics and pot experiments. GhSOD1-, GhAPX1-, and GhCAT1-overexpressing cotton showed higher tolerance to salinity than WT, and the synergistic effects of GhSOD1 and GhCAT1 were suggested to provide a new strategy for enhancing salt stress tolerance (Luo et al., 2013). Overall the results from transgenic approaches are complementary to the correlative evidence at the level of regulation of enzyme activity and of transcripts amounts and support the conclusion that enhanced antioxidant activity fosters salt acclimation. Figure 3 summarizes the various antioxidant systems in chloroplast, mitochondrion and peroxisome. It also indicates the sites of the safety valves and generator systems which will be discussed next, the alternative oxidase (AOX), the plastid terminal oxidase (PTOX), the respiratory burst oxidase homolog (RBOH) and the malate valve.

## Alternative oxidase (AOX)

Redox and ROS metabolism are tightly linked. Overreduction of electron transport chains enhances ROS production as discussed above. In higher plants, the respiratory electron transport chain (RET) feeds electrons to two terminal oxidases, namely cytochrome c oxidase (complex IV) for ATP synthesis and the cyanide-insensitive AOX for energy dissipation (Rogov et al., 2014). The conditional bifurcation to both pathways maintains the energy balance as well as redox homeostasis in dependence on the cellular energy demand. Drainage of electrons into the AOX pathway increases under stress (Zhang et al., 2016). Earlier the AOX capacity was demonstrated to respond to salinity in plants such as barley (Jolivet et al., 1990), soybean (Hilal et al., 1998) and carrot (Ferreira et al., 2008). Activation of AOX1a occurs via a thiol switch mechanism (Winger et al., 2007) and stimulates alternative respiration, decreases electron pressure and prevents over-reduction of the ubiquinone (UQ) pool which dampens excessive ${\text{O}}_{2}^{•-}$ and H_2_O_2_ generation and lowers ROS damage to plant cells under salt stress (Wang et al., 2010; Yoshida et al., 2011). In addition, AOX is involved in defining the threshold for the induction of programmed cell death (PCD) by signaling mechanism (Van Aken et al., 2009) and modulating the release of reactive nitrogen species (RNS). Release of nitric oxide (NO) in mitochondria is induced by accumulating reducing equivalents (Cvetkovska et al., 2014; Igamberdiev et al., 2014). In rice, expression of AOX1A and AOX1B in the nucleus is stimulated during saline conditions through mitochondrial retrograde regulation mediated by oxygen radicals (Li et al., 2013; Voss et al., 2013). Smith et al. (2009) reported AOX activation and stimulation of alternative electron transport in response to salinity stress. This mechanism allowed for suppressing ROS generation and increased the growth rates. Lower Na^+^ accumulation in shoots suggests a link between long distance Na^+^ transport and tissue ROS levels. This tentative dependency offers a mechanistic framework to exploit AOX and redox homeostasis to improve the acclimation ability to salt stress.

## Respiratory burst oxidase homologs (RBOH)

Homologs of NADPH oxidase in plants and animals contain cytosolic FAD- and NADPH-binding domains and six membrane-spanning helices. Two heme groups participate in the transfer of electrons from cytosolic NADPH or NADH to oxygen on the apoplastic side. This transfer produces ${\text{O}}_{2}^{•-}$ radicals in the extracellular matrix (Lambeth, 2004; Sagi and Fluhr, 2006). In plants, these enzymes are named RBOH and function in a plethora of processes, such as hyersensitive response to pathogens, abiotic stress tolerance, and local and systemic signaling (Torres et al., 2002; Monshausen et al., 2009). ROS released by RBOH act in several hormone-signaling pathways (Sagi and Fluhr, 2006).

RBOHA activity is induced by salt stress in the root elongation zone in Arabidopsis (Sagi and Fluhr, 2006). In Arabidopsis AtRBOHD and AtRBOHF are expressed in all plant organs and are the main isoforms involved in ROS-dependent regulation of Na^+^/K^+^ homeostasis under salt stress (Ma et al., 2012). AtRBOHD has also been demonstrated to mediate rapid systemic signaling triggered by multiple abiotic stresses (Miller et al., 2009) and to be required for salt acclimation signaling mediated by heme oxygenase HY1 in Arabidopsis (Xie et al., 2011). RBOHD shows the highest expression among the ten AtRBOH genes in *A. thaliana* (Suzuki et al., 2011) and functions in abscisic acid-dependent stomatal closure, flagellin-induced immune responses, and salt acclimation via ROS production (Torres et al., 2002; Pogány et al., 2009; Xie et al., 2011). Under salt stress RBOHD are clustered in the plasmamembrane and subjected to endocytosis and their activation facilitates the activation of redox signaling pathways and plays an important role in salt acclimation of Arabidopsis (Leshem et al., 2007; Xie et al., 2011; Hao et al., 2014).

Mild salt stress causes a rapid and transient accumulation of ROS in Arabidopsis peaking after 1 h followed by a second oxidative burst after about 6 h (Xie et al., 2011). The interpretation was that HY1 plays an important role in salt stress-signaling and that this pathway requires the participation of AtRBOHD-derived ROS from peak II. More recently, AtRBOHF was implicated in protecting shoot cells from transpiration-dependent accumulation of excess Na^+^ (Jiang et al., 2012). ROS generated by AtRBOHF have a specific role in regulating Na^+^ accumulation and soil-salinity tolerance (Jiang et al., 2012). Sakamoto et al. (2008) found that the expression of RBOHC/D/F genes is induced in response to salt stress in wild-type plants. The salt-responsive induction of *RBOH* accumulation was markedly suppressed in the *itn1-1* mutant. This mutant revealed increased tolerance to NaCl by affecting the ABA-mediated ROS production (Sakamoto et al., 2008). This suggest that the *itn1-1*, an ankyrin-repeat containing membrane protein, suppresses induction of the NADPH oxidase genes in response to salt stress, resulting in lower levels of H_2_O_2_, and that this may cause the salt-tolerant phenotype. The comparison of the salinity response of Arabidopsis wild type and *AtrbohD/F* double mutant indicates that the early H_2_O_2_ generation by NADPH oxidase triggers the antioxidant response in *A. thaliana*. The fortified antioxidant defense counteracts the subsequent ROS production and thereby mitigates the salt stress-derived injuries (Rejeb et al., 2014). According to Leshem et al. (2007), the salt-induced ROS production by NADPH oxidase in endosomes was suppressed in the phosphatidylinositol 3 kinase mutant (*pi3k*) and this mechanism causes a reduction in oxidative stress. In this experiment RBOH produces ${\text{O}}_{2}^{•-}$ which is coordinated by the phospholipid-regulated signaling pathway and takes part in signal transduction in response to salt stress (Leshem et al., 2007). RBOHs also contribute to proline accumulation in response to salt or mannitol stress (Rejeb et al., 2015b). The timing and the magnitude of RBOH-dependent ${\text{O}}_{2}^{•-}$-generation participate in inducing the salinity acclimation response on the one hand, but also in accelerating the deleterious effect of excessive ROS accumulation leading to salinity toxicity on the other hand.

RBOH-dependent ROS triggers signal transduction pathways and mediates local and systemic signaling (Miller et al., 2009; Marino et al., 2012). The initial Ca^2+^-influx through plasma membrane ion channels and the RBOH-mediated production of ROS are synergistically activated by the binding of Ca^2+^ to EF-hand motifs as well as Ca^2+^-dependent phosphorylation. Both mechanisms participate in regulating acclimation to salinity in plants including halophytes (Kurusu et al., 2015). RBOHs are central players in the Ca^2+^-ROS signaling network triggered by their phosphorylation during stress adaptation (Kimura et al., 2012; Gilroy et al., 2014). This Ca^2+^- and ROS-dependent signaling network regulates downstream events such as the Ca^2+^-dependent activation of the Na^+^/H^+^-exchangers SOS1 and NHX1, Na^+^ efflux from the cytosol, xylem loading of Na^+^, Na^+^ exclusion from leaves, induction of osmolyte synthesis and osmo-protective proteins, and overall the maintenance of cytosolic ion balance (Reguera et al., 2014). Work with *atrbohD/F* double mutants revealed that ROS are rate-limiting second messengers in ABA signaling. AtRBOHD/F function in ABA signal transduction in guard cell (Kwak et al., 2003). Following ABA perception in guard cells, active SnRK2 kinases such as OST1 (OPEN STOMATA 1) phosphorylate RBOHF and thereby stimulate ROS accumulation which in turn activates two MAPKs and regulates ABA-mediated stomatal closure (Danquah et al., 2014). RBOH-dependent ROS have been detected in vesicles in response to salt stress or during abscisic acid (ABA)-induced stomatal closure (Leshem et al., 2007). The different signaling mechanisms in RBOH activity control and the direct and indirect involvement in multiple downstream, processes characterize RBOH as a signaling hub for salinity acclimation.

## NADP-dependent malate dehydrogenase (NADP-MDH) and the malate valve

${\text{O}}_{2}^{•-}$ is generated in the PET by transfer of electrons from ferredoxin or reduced plastoquinone to O_2_, particularly if NADP^+^ or other terminal electron acceptors are unavailable. Likewise strong NADH feeding into the respiratory chain eases ${\text{O}}_{2}^{•-}$ generation as long as AOS is not activated. Thus, reoxidation of NADPH to NADP^+^ is important for balancing the ATP/NAD(P)H ratio and maintaining redox homeostasis. The photosynthesizing chloroplast employs diverse mechanisms to balance the rates of ATP and NADPH generation, e.g., by activating cyclic electron flow (CEF) which is under control of redox stimuli (Strand et al., 2015). Naturally such mechanisms do not allow for drainage of excess electrons if metabolic consumption is inhibited. Under such conditions activation of the malate-oxaloacetate (OAA) shuttle allows for transfer of reducing equivalents between cell compartments, e.g., under stress condition of plant (Heber, 1974; Taniguchi and Miyake, 2012; Figure 3). Another NADP^+^-generating mechanism is chloroplastic GR which reduces GSSG released in the water-water cycle. Excess electrons from photosynthetic electron transport are used by TRX-regulated NADP-dependent malate dehydrogenase (MDH) to reduce OAA to malate, thus regenerating the electron acceptor NADP^+^ (Scheibe et al., 2005). The resulting malate is subsequently translocated to the cytosol via the malate-OAA shuttle, where the interconversion of malate to OAA with concomitant reduction of NAD^+^ to NADH is catalyzed by the cytosolic NAD-MDH (Hara et al., 2006). The NADH is fed into the RET. This allows for maintenance of chloroplast redox homeostasis and plays an important role in the short-term adjustment of the NADP(H) redox state also in response to salinity stress (Scheibe et al., 2005). In line with this scenario, Cushman (1993) measured a more than twofold increase of chloroplast NADP-MDH transcript level in leaves of *Mesembryanthemum crystallinum* under salt stress. In a recent study, Gawronska et al. (2013) described a set of protective strategies which accompany acclimation to salinity in the halophytic species *M. crystallinum*. The malate valve appears to be of prime importance. Salinity stress increases activities of NAD-MDH in whole tissue extract, and specifically mitochondrial NAD-MDH and chloroplast NADP-MDH in salt tolerant rice cv CSR-1 and CSR-3 whereas the activities were inhibited in salt sensitive cultivars (Kumar et al., 2000). Apparently the malate valve-dependent redox balance constitutes an important mechanism in salt acclimation.

## Plastid terminal oxidase (PTOX)

The plastid terminal oxidase (PTOX) is a nucleus-encoded plastid-located plastoquinone (PQ)-O_2_ oxidoreductase (plastoquinol oxidase) which transfers electrons from PQ to O_2_ and forms H_2_O (Carol et al., 1999). It represents the key component of an alternative electron pathway which involves the reduction of PQ by NAD(P)H dehydrogenase (NDH) and the oxidation of reduced PQ by PTOX (Peltier and Cournac, 2002). PTOX is involved in chloroplast development and is suggested to act as safety valve to prevent the over-reduction of the photosynthetic machinery under stress conditions (Carol et al., 1999). However, additional features of PTOX have questioned the safety valve function owing to its ability to produce ROS under stress (Heyno et al., 2009; Feilke et al., 2014; Yu et al., 2014). When the PQ pool is highly reduced, PTOX itself produces superoxide (${\text{O}}_{2}^{•-}$) in a side reaction, triggering retrograde signaling to the cytosol and altering expression of response genes needed for acclimation to the environment (Yu et al., 2014; Krieger-Liszkay and Feilke, 2016). Overexpression of PTOX in *A. thaliana* did not protect against light-induced photodamage (Rosso et al., 2006) which appears contradictory to a protective function of PTOX. Under excess light PTOX overexpressors generate ${\text{O}}_{2}^{•-}$. If this ${\text{O}}_{2}^{•-}$ is efficiently detoxified by the antioxidant system, then even this mechanism may act as a safety valve (Heyno et al., 2009). If the antioxidant system is overwhelmed then PTOX-generated ${\text{O}}_{2}^{•-}$ would enhance damage formation. Overall, the function of PTOX appears to be Janus-like. PTOX-dependent ROS may damage the photosynthetic apparatus or contribute to its protection and regulation.

Up to 10% of the photochemically produced O_2_ in *Haematococcus pluvialis* was consumed by PTOX via the astaxanthin biosynthesis pathway. This pathway could lower the oxygen partial pressure and thereby reduce ROS release in the alga cell (Li et al., 2008). PTOX protects PSII by moving the site of ROS production from the appressed membranes with the majority of PSII to the nonappressed membranes where PTOX is located (Joët et al., 2002). In stressed plants, PTOX plays a regulatory role in carotenoid biosynthesis and in PQ oxidase activity in chlororespiration to re-oxidize reduced PQ (Bennoun, 1982; Kuntz, 2004; Campos et al., 2015). PTOX also controls the stromal redox poise (Trouillard et al., 2012). The abundance of PTOX is positively correlated with the intensity of salinity stress (Ivanov et al., 2012; Nawrocki et al., 2015). PTOX levels increase in salt stressed plants. PTOX-dependent electron drainage accounted for up to 30% of total PSII electron flow which provides strong evidence for its role as safety valve relative to photorespiration (Stepien and Johnson, 2009). Intensive H_2_O_2_ generation in photorespiration stimulated the activity of PTOX. This regulation seems to anticipate and subsequently counteract the effects of aggravating salinity stress. In a converse manner, inhibition of PTOX stimulated the H_2_O_2_ formation which might be important as signaling cue to initiate acclimation of halophytic *Thellungiella* plants (Wiciarza et al., 2015). PTOX attaches to the thylakoids at alkaline pH. This led to the suggestion that PTOX senses excessive alkalization of the stroma, subsequently attaches to the thylakoids and facilitates reoxidation of the PQ pool. By this mechanism overreduction of the photosynthetic electron transport is avoided and ROS generation decreased (Feilke et al., 2016).

## Other mechanisms of electron drainage under salinity

The accumulation of organic osmolytes, such as proline, glycine betaine, sugar alcohols, polyamines, and proteins e.g., from the late embryogenesis abundant (LEA) superfamily, in plasmatic compartments balances the osmotic potentials and maintains the low intracellular water potential of plants. Due to their compatible nature, these osmolytes counteract the harmful effects of ionic and osmotic stress (Verslues et al., 2006). Proline plays a crucial role in osmotic adjustment and acts as ROS scavenger, redox buffer, molecular chaperone which stabilizes proteins and membrane structures under stress (Matysik et al., 2002; Ashraf and Foolad, 2007). Proline synthesis via the glutamate pathway consumes 2 mol NADPH per mol proline and thus drains electrons from the chloroplast and buffers the cell reduction state (Hare and Cress, 1997). Accumulation of proline in leaves upon salt stress allows for continued carbon reduction and counteracts photoinhibition and excess ROS production. In the mitochondria proline is catabolized and the reducing power can be dissipated by RET coupled to AOX bypassing complex III and IV. Analyses of transcriptional regulation and knockout mutants indicate that the Arabidopsis Δ1-pyrroline-5-carboxylate synthetase1 (*P5CS1*) genes, the controlling step of proline synthesis, have clearly distinct functions. *P5CS1* is strongly induced under high salinity (Szekely et al., 2008). Consistent with the upregulation of *AtP5CS1, p5cs1* knockout mutants have greatly reduced proline levels during salt stress, resulting in reduced growth and altered ROS levels, suggesting that they are hypersensitive to salt (Szekely et al., 2008). Similar to proline, glycine betaine is an organic osmolyte synthesized by several plant families to balance the osmotic potential of intracellular compartments (Chen and Murata, 2011) under salinity. The plant pathway of synthesis consumes two electrons as net balance (Sakamoto and Murata, 2000). Activation of antioxidant mechanisms by proline and glycine betaine during salinity has been studied using tobacco bright yellow-2 suspension cultured cells (Hoque et al., 2007; Banu et al., 2009). Salinity significantly decreased the levels of reduced ascorbic acid and GSH, and the activity of water-water-cycle enzymes, and exogenous application of proline or glycine betaine increased the activity of these enzymes (Hoque et al., 2007). These results suggest a role of proline and glycine betaine in the regulation of antioxidant enzymes during salinity.

The last example of electron drainage with major significance for salinity is the pathway of photorespiration. Salinity-induced stomatal closure in moderately salt-stressed leaves leads to a drop in intercellular CO_2_ concentration, increased oxygenation reaction of ribulose-1,5-bisphosphate carboxylase/oxygenase, enhanced formation of photorespiratory metabolites such as phosphoglycolate, glycine and serine, and concomitant increase in H_2_O_2_ release in the peroxisomes, and CO_2_ and NADH in the mitochondrion (Di Martino et al., 1999). The maintenance of electron transport by photorespiration in CO_2_-free air corroborates the significance of photorespiration in salt-stressed leaves (Di Martino et al., 1999). Sustained rates of electron transport due to photorespiration and the formation of zeaxanthin during salt stress probably mitigate photoinhibitory damage (Sharma and Hall, 1992). The xanthophyll cycle is known as dynamic photoinhibition process that prevents overexcitation of the photosynthetic apparatus by dissipation of excess excitation energy (Krinsky, 1989; Niyogi et al., 1998). But, photorespiration also releases H_2_O_2_ in the peroxisomes as outlined above. The antioxidant system in the peroxisome efficiently detoxifies the photorespiratory H_2_O_2_. Jiménez et al. (1997) reported the presence of APX and MDHAR in peroxisomal membranes and argued that the membrane-bound antioxidant enzymes protect against H_2_O_2_ leaking out of peroxisomes. The photorespiratory NADH can be used in complex IV dependent RET for ATP synthesis or dissipated by AOX. This section on alternative drainage mechanisms only provides examples of involved pathways and cannot provide a comprehensive view.

## Conclusions and outlook

**Figure 4:**
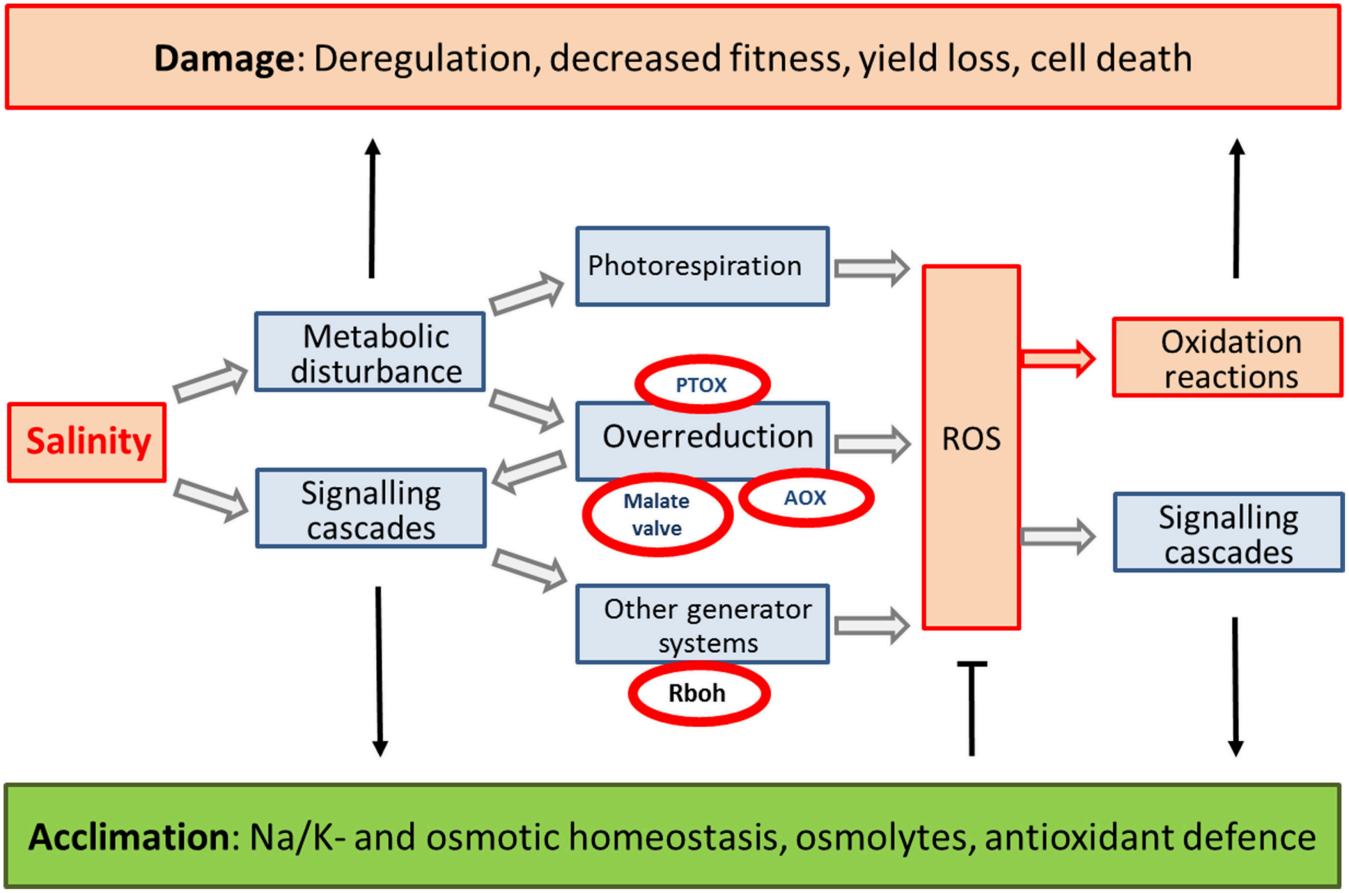
**Circuitry of redox and ROS-related events in salinity stress response**. Salinity stress causes metabolic imbalances and activates signaling pathways. The metabolic imbalances increase the ROS generation e.g., by enhanced photorespiration or cause overreduction of PET and RET as described in the review. The latter is modulated by AOX, PTOX and malate valve. Signaling activates RBOH. Accumulating ROS either cause excessive oxidation reactions leading to damage or via redox- and ROS-dependent signaling and regulation allow for proper acclimation.

Maintenance of redox homeostasis is central to plant survival under salinity stress. Successful acclimation to saline growth conditions involves control of generation systems and tuning of antioxidant mechanisms. Under normal growth conditions generation and scavenging of ROS, and repair of damage are balanced. Salinity interferes with metabolism by ionic and osmotic effects and alters the redox and ROS state of the cell (Figure 4). This review demonstrates the flexible adjustment of each of the steps in response to salinity. It is striking that the described redox and ROS-related mechanisms of defense under salinity fit to the defense repertoire under other stresses such as photooxidative conditions. The specificity comes from the qualitative and quantitative use of stress-specific isoforms as discussed above. The principle differences between salt-sensitive and -tolerant genotypes includes specific symptomatic differences in redox and ROS generation. The central mechanisms realizing salt acclimation within the given tolerance range is selective short and long distance ion transport, safe ion compartmentation, synthesis of compatible solutes, and adjustment of osmotic homeostasis. As long as these mechanisms realize effective ion detoxification, deregulation of redox and ROS homeostasis is a minor component in stress acclimation. This changes if the salinity stress approaches the tolerance limits or during transition periods. Then the ultimate reason for decreased growth, reduced fitness and finally cell death are alteration in metabolism and excessive ROS accumulation (Figure 4). Some environmental conditions enhance salinity stress like periodical flooding with sea water, drought in saline environment or irrigation with saline water. The various mechanisms of dissipation of excess reducing power are mutually dependent. *A. thaliana* lacking chloroplast malate dehydrogenase are phenotypically inconspicuous (Hebbelmann et al., 2012). Proline synthesis, increased photorespiration and activation of thiol peroxidase appear to compensate for the deficiency in NADP-MDH in high light treated *nadph-mdh* plants. This example shows the flexibility and partial redundancy of processes to control ROS release due to excessively accumulating reducing power and control of antioxidant defense which also contributes to salt stress acclimation. As long as the homeostasis mechanisms are functional and control the metabolic imbalances and keep ROS and redox deviations under control, small changes in redox- and ROS signatures are used to control gene expression, protein synthesis, metabolic activities and enable acclimation.

## Author contributions

All authors listed, have made substantial, direct and intellectual contribution to the work, and approved it for publication.

### Conflict of interest statement

The authors declare that the research was conducted in the absence of any commercial or financial relationships that could be construed as a potential conflict of interest.

## Abbreviations

AOX: alternative oxidase
APX: ascorbate peroxidase
CAT: catalase
CEF: cyclic electron flow
DHAR: dehydroascorbate reductase
GPX: glutathione peroxidase
GR: glutathione reductase
GSH: glutathione
GST: glutathione-S-transferase
MDH, malate dehydrogenase: NADP- or NAD-dependent
MDHAR: monodehydroascorbate reductase
PET: photosynthetic electron transport
PRX: peroxiredoxin
POD: guaiacol heme peroxidase
PTOX: plastid terminal oxidase
RBOH: respiratory burst oxidase homologue
RET: respiratory electron transport
ROS: reactive oxygen species
SOD: superoxide dismutase
TRX: thioredoxin.
